# Intravenous analgesia with ultra-high-dose morphine for the treatment of headache and successful withdrawal of morphine: A case report and literature review

**DOI:** 10.1097/MD.0000000000022919

**Published:** 2020-10-30

**Authors:** Xiaoling Zhang, Jialei Zhang, Yunyi Du, Mei Wang, Yangjun Gao, Lurong Zhou, Jing Lu, Jun Zhao

**Affiliations:** a Department of Oncology, Changzhi People's Hospital; b Department of Anesthesiology, Changzhi People's Hospital; c Quality Control Department, Changzhi People's Hospital, Changzhi, Shanxi; d Department of Pathophysiology, College of Basic Medical Sciences, Zhengzhou University; e Collaborative Innovation Center of Henan Province for Cancer Chemoprevention, Zhengzhou, Henan, China.

**Keywords:** cancerous pain, morphine withdrawal, ultra-high-dose morphine

## Abstract

**Rationale:**

Pain is the fifth vital sign of human beings. Morphine is the first choice for relieving moderate to severe cancer pain. Most of the previous studies merely focused on the analgesic effect of high-dose or ultra-high-dose morphine in patients with advanced cancers but did not report any cases related to successful morphine withdrawal.

**Patient concerns:**

A 42-year-old woman was admitted to our hospital in March 2019.

**Diagnosis:**

She was diagnosed with progressive aggravation of headache for 1 month, which was meningeal metastasis of lung cancer.

**Interventions:**

Symptomatic treatments like dehydration, hormone, intrathecal injection chemotherapy and an increased dose of osimertinib to 160 mg/day were applied but showed poor curative effects. The patient refused whole-brain radiotherapy. Pain intensity level was re-evaluated and the patient scored 9 based on numerical rating scale, which suggested that the patient suffered from severer cancerous pain. Thus, the patient started to receive morphine for treating headache.

**Outcomes:**

The patient's headache was alleviated after receiving high-dose morphine treatment, and she continued to undergo anti-cancer treatment. After tumor remission, the patient's morphine dose gradually decreased and eventually stopped, without any withdrawal symptoms. In addition, the quality of life of the patient was greatly improved with performance status scored 2 and limb muscle strength increased from Grade 2 to Grade 5.

**Lessons:**

For patients with advanced cancers, the application of ultra-high-dose morphine may significantly relieve cancerous pain, improve survival and quality of life, and overcome their fear for death and desperation, which contributes to the establishment of a basis for subsequent anticancer treatments. Thus, timely effective pain management and routine anticancer treatments are the key to addressing the cancer pain problem.

## 1 Introduction

Pain is the fifth vital sign of human beings. Statistically, ∼25% of newly diagnosed cancer patients suffer from cancerous pain. Moreover, the incidence rate rises to 60% to 80% in patients with advanced cancers, among which one third of patients experience severe pain.^[1]^ Morphine is the first choice for relieving moderate to severe cancer pain.^[2,3]^ Most of the previous studies merely focused on the analgesic effect of high-dose or ultra-high-dose morphine in patients with advanced cancers but did not report any cases related to successful morphine withdrawal. This study reported a case of a patient with meningeal metastatic lung cancer who was administrated ultra-high-dose morphine (the patient received injection of 480-mg morphine daily, which was equivalent to 1440 mg of oral morphine) for treating headache. After achieving efficient pain relief by morphine, the patient continued her anticancer therapies and cancerous pain was significantly reduced. Then morphine was gradually withdrawn without inducing any withdrawal symptoms. In addition, the quality of life of the patient was greatly improved with performance status (PS) scored 2 and limb muscle strength increased from Grade 2 to Grade 5.

## 2 Case presentation

A 42-year-old woman was admitted to our hospital in May 2017 due to progressive aggravation of lumbodorsal pain for 1 month. The patient was diagnosed with peripheral lung adenocarcinoma in the apicoposterior segment of superior lobe of left lung (cTxNxM1, quantification type IV, EGFR 19del) with multiple bone metastases in the fifth, seventh, and twelfth thoracic vertebrae and the second and fourth lumbar vertebrae. Herein, the patient was orally administrated 250 mg of gefitinib once daily and injected with zoledronic acid. In March 2018, the patient complained about head distention and ache. Results of peripheral blood genetic test showed T790M mutation. Thus, the patient was orally administrated 80 mg of osimertinib once daily and headache was found to be effectively relieved. In October 2018, the patient reported a relapse of headache and was diagnosed with meningeal metastases by pathological examination of cerebrospinal fluid. Hence, the patient received intrathecally injection of 10-mg methotrexate and 5-mg dexamethasone for three times and was administrated an increased dose of osimertinib to 160 mg/day.^[4]^ Headache symptom was alleviated after the treatment. However, headache returned to the patient after 2 months and further intensified. The patient refused whole-brain radiotherapy (WBRT) but accepted pemetrexed as a chemotherapy for 1 week. Headache was relieved after the treatment. Then, the patient herself discontinued routine chemotherapy. In March 2019, the patient reported an aggravation of headache symptom, which was improved after dehydration therapy. Then, the patient was administrated pemetrexed and carboplatin as chemotherapies in combination with a single intrathecal injection. One week later, the patient developed a sudden severe headache with general numbness, dysphoria, projectile vomit, and decreased limb muscle strength to Grade 1 or Grade 2. Pain intensity of the patient scored 7, which suggested severe pain according to the numerical rating scale (NRS). Physicians speculated that pain could be caused by elevated intracranial pressure associated with meningeal metastases. Symptomatic treatments like dehydration and hormone therapies were applied but showed poor curative effects. Pain intensity level was re-evaluated and the patient scored 9 based on NRS, which suggested that the patient suffered from severer cancerous pain. Thus, the patient started to receive morphine treatment.

### 2.1 Morphine treatment

#### 2.1.1 Dose titration during morphine intolerance

The U.S. Food and Drug Administration identifies opioid tolerance as receiving 25 mcg/h fentanyl patch, at least 60 mg of morphine daily, at least 30 mg of oral oxycodone daily, at least 8 mg of oral hydromorphone daily, 25 mg of oxymorphone daily, or an equivalent dose of another opioid for a week or longer; otherwise, it may be classified as opioid intolerance.^[5]^ Accordingly, the patient was defined as opioid-naïve before receiving morphine treatment in this study. According to the “Guidelines for Diagnosis and Treatment of Cancer Pain (2018 Edition)” issued by the National Health Commission of the People's Republic of China (hereinafter referred to as the guidelines), all doses should be titrated to appropriate effect. On this basis, the patient with severe cancerous pain received hypodermic injection of 5-mg morphine on March 12, 2019 (Day 1, the first day of morphine treatment). The doses of morphine extended-release tablet were adjusted based on the doses of morphine immediate-release tablet that were used for relieving breakthrough pain (BTP) occurring in the initial 24 h. The dosage of morphine extended-release tablet reached 660 mg (330 mg Q12 h) on the sixth day of morphine treatment (Table 1).

**Table 1:**
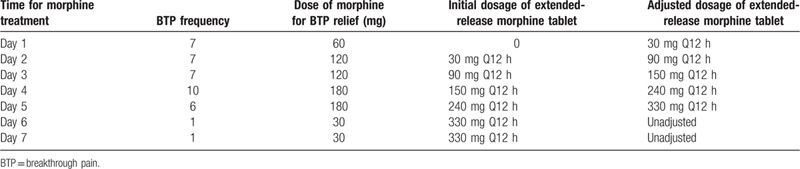
Dose titration during morphine intolerance.

#### 2.1.2 Pain management after morphine tolerance by multi-disciplinary team

The dose of morphine reached a peak on the sixth day of treatment. Morphine-caused adverse effects occurred in the patient, including general numbness, decreased limb muscle strength, uroschesis, nausea, emesis, and pruritus. Based on the patient's condition, our hospital held a multi-disciplinary team (MDT) consultation in order to determine superior therapeutic strategies that could achieve favorable analgesic effects as well as reduce adverse effects. In view of the poor condition of the patient, radiologists recommended WBRT if intracranial pressure could be reduced after curative care. Neurosurgeons refused ventriculo-peritoneal (pleural) shunt because of meningeal metastatic lung cancer. Pain clinicians recommended establishing an intrathecal drug delivery system by lumbar catheterization to decrease the dose of morphine. Neurologists noticed that the intracranial pressure of the patient reached 500 to 550 mm H_2_O and thereby speculated that anticancer treatments could be the key to pain relief for that the increase in intracranial pressure could be mainly attributed to direct stimulation by tumor cells or pain related to meningeal metastases. According to oncologists, an oral dose equivalent to 10% to 20% of total morphine taken in the initial 24 h should be administrated once BTP occurred for effectively relieving pain and improving the quality of life of the patient. The patient had taken 30 mg of oral morphine for 1 week, which made her a morphine-tolerant patient. Besides, the patient did not receive routine chemotherapy or antiangiogenic therapy. Thus, the oncologists recommended performing pemetrexed and bevacizumab combination treatment and closely monitoring the adverse effects of morphine. After serious consideration and discussion, the patient and her dependents refused lumbar cistern catheterization but agreed to receive anticancer treatment 1 week later.

#### 2.1.3 Intravenous infusion of ultra-high-dose morphine for relieving cancer pain and successful morphine withdrawal after anticancer treatment

The patient developed frequent vomit, which caused an inaccuracy in oral morphine intake. According to the National Comprehensive Cancer Network Guidelines for Adult Cancer Pain, oral morphine can be conversed into intramuscular or intravenous at a conversion ratio of 3:1. Hence, in order to maintain a stable plasma concentration, reduce adverse effects of oral morphine and maximize pain relief, the patient began to receive 240 mg/d morphine via infusion pump since Day 22. The doses of morphine were dynamically adjusted according to the guidelines. The dose of morphine used on Day 28 was increased to 480 mg/day. The clinical signs of the patient and adverse effects were continuously observed and results showed that cancerous pain was significantly relieved and no recurrence of BTP was observed. In addition to several adverse effects like nausea, vomit and constipation, no toxic symptoms such as dizziness, respiratory depression were observed.

**Figure 1:**
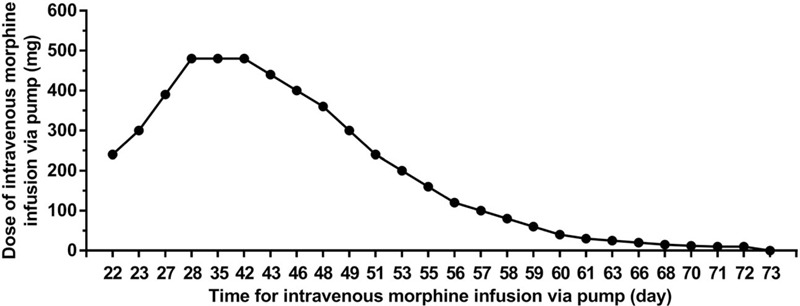
The processes of intravenous analgesia with ultra-high dose of morphine and drug withdrawal.

On Day 42, the patient developed lethargy with mild nausea. Considering that the patient was experiencing unmanageable adverse effects and cancerous pain had been partially relieved by anticancer treatment, opioid dose reduction was warranted for alleviating the adverse effects caused by opioids. As described in the guidelines, the dose should be gradually tapered down. The analgesic dose may be reduced by 10% to 25% daily until the dose is equivalent to 30 mg of oral morphine for ameliorating opioid-related withdrawal symptoms. The effective dose above may be continued for 2 days and then withdrawn. Accordingly, the dose of morphine was reduced once every 2 days since Day 43. By Day 71, the dosage of morphine had been adjusted to intravenous infusion of 10 mg/d morphine. This morphine dose was continued for 2 days and then withdrawn on Day 73 (Fig. 1). Two-week follow-up showed no symptoms of morphine withdrawal. Re-evaluation of the condition of the patient demonstrated that limb muscle strength was enhanced to Grade 5 and no BTP occurred. The patient began routine anticancer treatment.

## 3 Discussion

World Health Organization (WHO) identifies morphine consumption as one of the golden criteria used for evaluating pain management. Morphine is characterized by no ceiling effect, which means that morphine doses may be unlimitedly adjusted on the basis of pain intensity levels. According to Edmonton Classification Scale, the doses of oral morphine include: regular doses (<300 mg/d), high doses (300–600 mg/d), and ultra-high doses (>600 mg/d).^[6]^ Although with treatments under the guidance of the three-step “ladder” for cancer pain relief developed by WHO, ∼10% to 20% of patients still developed refractory cancer pain, among which 5% to 8% of patients required high doses of opioids. According to the statistics in a previous retrospective study, cancer patients receiving ultra-high-dose morphine treatment accounted for only 1.6%.^[3,7]^ Berevitch et al retrospectively analyzed the medical records of 651 patients hospitalized in Tel Hashomer Hospital between January 1, 1996 and December 31, 1997, and concluded that 453 patients received regular doses of morphine as the first-line therapy. A total of 55 patients took doses higher than 299 mg/d, among which 19 patients received doses between 300 and 599 mg/d and the rest received doses higher than 599 mg.^[8]^ Plentiful previous publications reported the application of high-dose or ultra-high-dose morphine for pain relief in patients with advanced cancers. However, the patients recruited all suffered from advanced cancers with poor prognoses and short survivals. Thus, none of these studies addressed the survival issue of patients receiving ultra-high-dose morphine or reported any cases of successful withdrawal from ultra-high-dose morphine. Berevitch et al compared the survivals between patients receiving regular doses of morphine and those receiving high doses, and found that the mean survival periods of patients receiving morphine with regular doses (<300 mg/d), high doses (300–599 mg/d), or ultra-high doses (>599 mg/d) were 14 days, 15 days, and 13 days, respectively. There were no significant differences among these groups.^[8]^ In this study, the patient received ultra-high doses of morphine via intravenous morphine infusion pump based on the comprehensive assessment results for the patient's condition. The doses of morphine were tapered down to withdrawal after cancerous pain had been significantly reduced by anticancer treatment. Follow-up results showed that no symptoms of withdrawal occurred. The quality of life of the patient was greatly improved with PS scored 2 and limb muscle strength elevated from Grade 2 to Grade 5. Altogether, these results suggested that effective pain management may be a prerequisite for anticancer interventions, which in turn determine the withdrawal of analgesics. Thus, pain management is necessary for patients with advanced cancers to continue their anticancer treatments for that effective pain control not only reduces cancerous pain in patients but also strives for the precious time for further anticancer treatments.

Lu et al^[9,10]^ demonstrated that the combination therapy involving intrathecal morphine pump and wireless-controlled analgesic pump achieved favorable analgesic effects in patients with advanced cancers. Programmable morphine pump, also known as intrathecal drug delivery system, is an efficient analgesic device for pain management by continuously delivering low-dose morphine. The pump is filled with morphine and implanted under the covering of the abdominal muscles of patients. A small catheter is inserted into the subarachnoid space and is threaded upward. Then the catheter is tunneled under the skin to the abdomen and is connected to the pump. The most distinct advantage of morphine pump is that it can exert an equivalent pain-relieving effect to oral morphine with a much lower dose. However, the patient refused morphine pump implantation after fully communicating with physicians. Instead, the patient received morphine treatment on ultra high doses for 73 days. Initially, several adverse effects such as nausea, vomit, dizziness, pruritus, uroschesis, constipation, and decreased limb muscle strength of limbs occurred in the morphine-intolerant patient, whereas the symptoms were gradually relieved and disappeared with the development of morphine tolerance, except for constipation. Morphine doses were titrated to appropriate effect according to the guidelines, and results showed that no symptoms of morphine overdose (such as respiratory depression) occurred in the morphine-intolerant patient. The withdrawal of morphine should obey the principle of “slow-rapid-slow.” Accordingly, in this report, ultra-high-dose morphine was gradually withdrawn without triggering any symptoms of withdrawal, which suggested that no morphine addiction was developed in the morphine-tolerant patient in spite of ultra-high-dose morphine treatment. These results consistently indicated the safety of ultra-high-dose morphine in the management of cancer pain. However, more clinical trials should be performed for further verifying the current findings.

In conclusion, although ultra-high dose of morphine has not been widely used in clinical practices, it may be generally considered safe on the basis of strict application ranges and close monitoring of adverse reactions. For patients with advanced cancers, the application of ultra-high-dose morphine may significantly relieve cancerous pain, improve survival and quality of life, and overcome their fear for death and desperation, which contributes to the establishment of a basis for subsequent anticancer treatments. Thus, timely effective pain management and routine anticancer treatments are the key to addressing the cancer pain problem.

## Author contributions

**Conceptualization:** Xiaoling Zhang, Jialei Zhang, Jing Lu, Jun Zhao.

**Data curation:** Yunyi Du.

**Investigation:** Mei Wang.

**Resources:** Yangjun Gao.

**Software:** Lurong Zhou.

**Writing – original draft:** Xiaoling Zhang, Jialei Zhang.

**Writing – review & editing:** Jing Lu, Jun Zhao.
